# Latent Infection of *Valsa mali* in the Seeds, Seedlings and Twigs of Crabapple and Apple Trees is a Potential Inoculum Source of Valsa Canker

**DOI:** 10.1038/s41598-019-44228-w

**Published:** 2019-05-23

**Authors:** Xiang-long Meng, Xing-hua Qi, Ze-yuan Han, Yong-bin Guo, Ya-nan Wang, Tong-le Hu, Li-ming Wang, Ke-qiang Cao, Shu-tong Wang

**Affiliations:** College of Plant Protection, Hebei Agricultural University, Baoding, 071001 P.R. China

**Keywords:** DNA, High-throughput screening, Fungal pathogenesis

## Abstract

A real-time quantitative PCR assay using a species-specific primer pair was developed to rapidly and accurately quantify *Valsa mali*, the causative pathogen of apple Valsa canker (AVC), in crabapple seeds, crabapple seedlings, apple twigs and apple seeds. Surveys were conducted in different regions, and crabapple or apple seeds were collected for *V*. *mali* detection by qPCR assay. Our results showed that 12.87% to 49.01% of crabapple seeds collected from different regions were positive for *V*. *mali*. The exopleura and endopleura were the two major areas of *V*. *mali* infection in crabapple seeds. The presence of *V*. *mali* infection in crabapple seeds was also confirmed by a high-throughput sequencing approach. With the growth of crabapple seedlings, the concentration of *V*. *mali* gDNA in crabapple seedlings gradually increased until eight or more leaf blades emerged. One-year-old twigs from an apple scion nursery were infected with *V*. *mali*, and only apple seeds from infected apple trees showing evident Valsa canker symptoms carried *V*. *mali*. In conclusion, this study reports that crabapple seeds and apple seeds carried *V*. *mali* as latent inoculum sources. *V*. *mali* infected not only apple tissues but also crabapple seedlings, which are the rootstocks of apple trees. This study indicated that the inoculum sources for AVC vary. Application of a novel qPCR assay can potentially improve the accuracy of early diagnosis, and is helpful to reveal the epidemic regularity of AVC.

## Introduction

*Valsa mali* Miyabe & Ymada (anamorph *Cytospora sacculus*), the pathogen that causes apple Valsa canker (AVC), is widely spread in eastern Asia^1–3^ and causes serious losses to apple production, especially in China^4^. China is the largest producer of apples; however, the average incidence of AVC is approximately 52.7%, which severely limits the development of the apple industry^5^. Control of this disease is difficult because the pathogen can infect the phloem and xylem of the host tissue. Thus, conventional fungicide treatments may not effectively access and inhibit the pathogen inside the plant tissues.

The AVC pathogen infects apple trees through the wounds of organ surfaces, such as fruit scars, frost injuries and fresh pruning wounds^6^. Trunks, twigs and scaffold limbs are the primary infected tissues, appearing with distorted, swollen, sunken, or cracked bark covered with red pustules^3^. In addition, the fungal fruiting bodies, such as pycnidia, are another feature of this pathogen, as the conidia with spiral, orange-coloured tendrils extend from the canker bark^7^. With the development of infection, apple canker symptoms gradually extend, causing the infected organs to lose their physiological activities, and eventually resulting in the death of the entire tree.

In addition to the typical symptoms of AVC, in some cases, latent infections by the pathogens can also exist in apple tissues^8^, which could be a potential risk of canker disease in apple trees, especially in newly established apple orchards. Latent infection usually lasts a long period of time, until the apple trees suffer a severely unfavourable environment and then show symptoms. The latent infections of *V*. *mali* serve as a source for the outbreak of the disease in the new apple orchards in China. In most apple production processes in China, apple twigs are usually grafted onto rootstocks to maintain good quality. Thus, the infected grafted twigs and rootstocks without typical symptoms might be the primary source of infection in newly planted orchards. However, this hypothesis is still difficult to confirm because of the difficulty of detecting *V*. *mali* in symptomless tissues. A rapid and accurate method was desired to be developed to identify the source of *V*. *mali* infection, which can be used to assist in the reduction of t the primary inoculum and the development of disease control strategies^9^.

Thus far, traditional methods, such as spore and visual symptom observations, are still the main approaches to identify the fungal AVC pathogen. However, these methods are time consuming and labour intensive. With the development of molecular techniques, PCR based on sequence analysis has been demonstrated to be an effective method for the detection, identification and classification of plant pathogens^10^. A nested PCR assay was developed to detect *V*. *mali* on symptomless and symptomatic apple tissues, and the accuracy of nested PCR for detection of the pathogen from the symptomless samples was relatively high^11^. However, this method could not be used to quantify the amount of pathogen in apple plants.

Real-time quantitative PCR assays seem to be a good choice to detect and quantify the amount of pathogen due to the advantage of sensitivity, reliability, rapidity and quantitative results^12^. This method has been widely used to detect and quantify many kinds of plant pathogens in different hosts, such as viruses^13^, bacteria^14,15^ and fungi^16^. Using the Ct values from the qPCR assay, this method can quantify trace amounts of target DNA, which is suitable for quantifying *V*. *mali* in latent infections in apple trees. However, to the best of our knowledge, this method has not been developed to detect and quantify the AVC pathogen.

The objectives of this study were to (i) develop a species-specific qPCR assay to rapidly and accurately identify and quantify *V*. *mali*; (ii) employ the qPCR assay to detect the AVC pathogen from crabapple seeds, crabapple seedlings, apple seeds and apple seedlings; and (iii) evaluate the inoculum sources for new and old apple orchards.

## Results

### Primer specificity test

The primers were specific for the amplification of the DNA of sixteen *V*. *mali* strains, and non-specific for twenty-two other species of fungal strains, including the closely related species *V*. *mali* var. *pyri*. The Ct values of seventeen *V*. *mali* strains, including the *V*. *mali* var. *pyri* strain, were between 19.4 and 26.57, while non-specific DNA amplifications from the reference strains were not observed when using 10 ng DNA (Table 1). The amplification products of the *V*. *mali* var. *mali* strains showed 100% identity to the sequences of the EF-1α gene for *V*. *mali* strains in the GenBank database.

**Table 1:** Isolates of *Valsa mali* and other fungal species used to determine the specificity of species-specific primers in the real-time qPCR assay.

No.	Species	Isolate code	Host tissue	Region^a^	Ct^b^
1	*Valsa mali* var. *mali*	03–8	Apple twigs	SX	22.31 ± 0.01
2	*V*. *mali* var. *mali*	Vmm31	Apple twigs	LQSX	19.4 ± 0.13
3	*V*. *mali* var. *mali*	Vmm466	Apple twigs	JCGS	22.89 ± 0.04
4	*V*. *mali* var. *mali*	Vmm463	Apple twigs	CWSX	23.35 ± 0.04
5	*V*. *mali* var. *mali*	Vmm462	Apple twigs	JCGS	26.57 ± 0.02
6	*V*. *mali* var. *mali*	Vmm459	Apple twigs	BXSX	25.89 ± 0.07
7	*V*. *mali* var. *mali*	Vmm458	Apple twigs	JCGS	22.77 ± 0.15
8	*V*. *mali* var. *mali*	Vmm440	Apple twigs	BDHB	23.93 ± 0.01
9	*V*. *mali* var. *mali*	Vmm320	Apple twigs	XTHB	24.88 ± 0.08
10	*V*. *mali* var. *mali*	Vmm318	Apple twigs	XTHB	23.69 ± 0.13
11	*V*. *mali* var. *mali*	Vmm313	Apple twigs	TSHB	20.63 ± 0.15
12	*V*. *mali* var. *mali*	Vmm309	Apple twigs	TSHB	20.18 ± 0.3
13	*V*. *mali* var. *mali*	Vmm289	Apple twigs	TSHB	24.12 ± 0.1
14	*V*. *mali* var. *mali*	Vmm277	Apple twigs	CZHB	21.97 ± 0.29
15	*V*. *mali* var. *mali*	Vmm263	Apple twigs	CZHB	25.03 ± 0.01
16	*V*. *mali* var. *mali*	Vmm250	Apple twigs	ZJKHB	23.43 ± 0.18
17	*V*. *mali* var. *pyri*	Vmp-1	Pear twigs	SX	26.58 ± 0.12
8	*Acremonium* spp.	ZDB-1	Apple fruit	SJZHB	—
19	*Acremonium* spp.	ZDB-2	Apple fruit	TSHB	—
20	*Alternaria mali*	QY-2	Apple leaves	BDHB	—
21	*Alternaria mali*	SJZ-1	Apple leaves	SJZHB	—
22	*Alternaria* spp.	A7	Apple fruit	XTHB	—
23	*Alternaria* spp.	A6	Apple fruit	SJZHB	—
24	*Aspergillus niger*	HQM-1	Apple twigs	BDHB	—
25	*Botryosphaeria dothidea*	XTFS	Apple twigs	XTHB	—
26	*Botryosphaeria dothidea*	WDFS	Apple twigs	BDHB	—
27	*Botryosphaeria dothidea*	HDFS	Apple twigs	HDHB	—
28	*Cercospora* spp.	W2	Apple fruit	TSHB	—
29	*Cercospora* spp.	W1	Apple fruit	TSHB	—
30	*Alternaria* spp.	TJ	Apple leaves	BDHB	—
31	*Fusarium oxysporum*	FO-1	Apple roots	BDHB	—
32	*Fusarium oxysporum*	FO-2	Apple fruit	BDHB	—
33	*Fusarium oxysporum*	FO-3	Apple fruit	BDHB	—
34	*Fusarium proliferatum*	FP-1	Apple roots	BDHB	—
35	*Penicillium* spp.	QM-1	Apple twigs	BDHB	—
36	*Trichoderma* spp.	MM-1	Apple twigs	BDHB	—
37	*Trichothecium roseum*	TRHB-1	Apple fruit	TSHB	—
38	*Trichothecium roseum*	TRHB-2	Apple fruit	HDHB	—

^a^SX represents strains isolated from Shanxi Province; LQSX, CWSX and BXSX represent strains isolated from Liquan city, Changwu city and Binxian city in Shanxi Province, China; JCGS represents strains isolated from Jingchuan city Gansu Province; and BDHB, XTHB, TSHB, CZHB, ZJKHB, SJZHB and HDHB represent strains isolated from Baoding city, Xingtai city, Tangshan city, Cangzhou city, Zhangjiakou city, Shijiazhuang city and Handan city in Hebei Province, China. Each sample was repeated three times.

^b^Ct values for 10 ng genomic DNA; “-” indicates that no amplification was detected.

### Sensitivity and standard curve for qPCR

**Figure 1:**
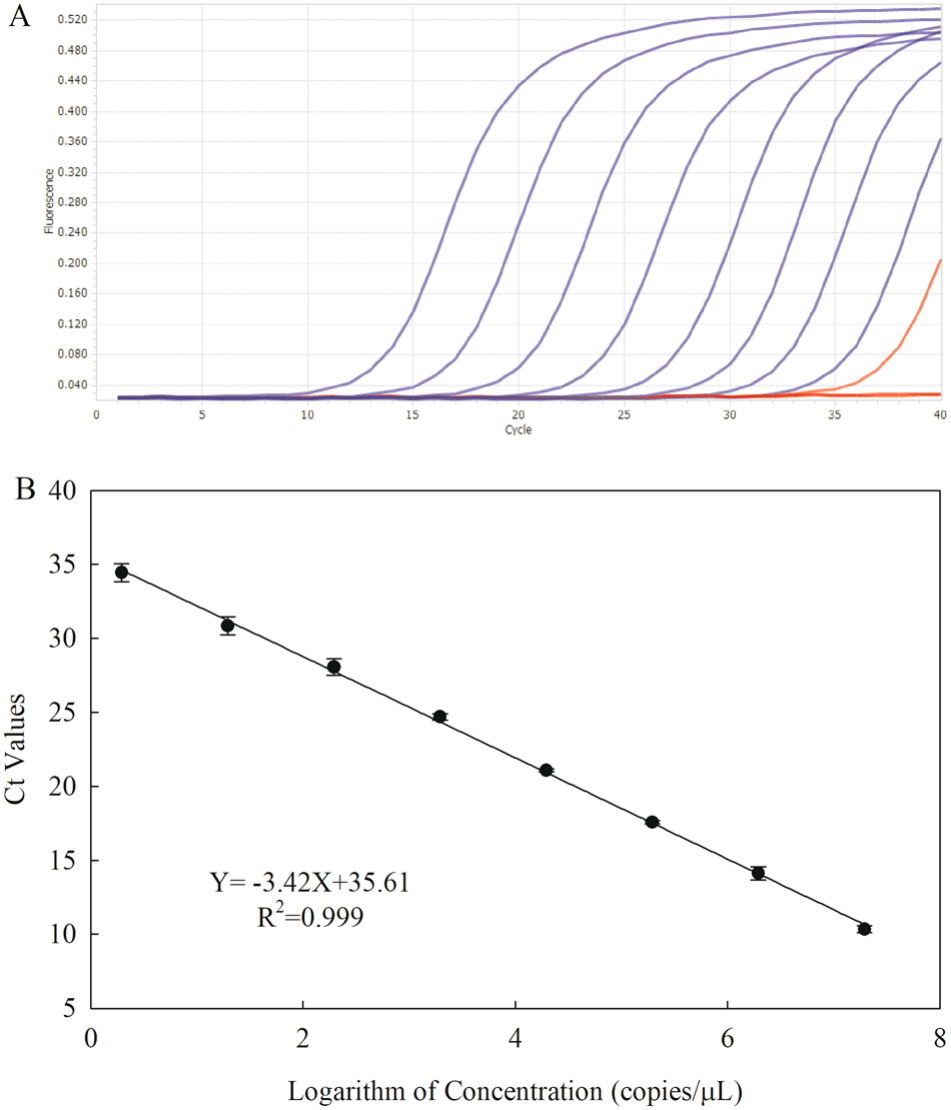
The amplification curve (A) and standard curve of qPCR for gradient dilution of *V*. *mali*. genomic DNA. (**A**) The amplification curve represents the DNA with the concentrations ranging from 264 ng µL^−1^ to 0.264 fg µL^−1^. (**B**) The log amount of the sample copy number calculated by the DNA concentration was plotted versus the Ct value, and the equation of the regression line and the correlation coefficient (*R*^2^) are displayed in the graph. The standard curve was generated using gradient dilution genomic DNA corresponding to 1.95 to 1.95×10^7^ *V*. *mali* strain sample copies per microlitre. Each concentration of DNA was repeated three times. Error bars represent standard deviation from three replicate reactions.

The standard sample of *V*. *mali* gDNA with a concentration of 2 copies per reaction volume produced replicates with a CV = 32.6. A lower concentration of *V*. *mali* gDNA (0.2 copies/µl) produced replicates with a CV > 35. Hence, the limit of quantification was 2 genome copies per reaction, where the corresponding Ct value was 34.4. A standard curve was generated to correlate the amplification signal with cell quantities. Ct values of a 10-fold DNA dilution series of a *V*. *mali* strain as the ordinate were plotted against the logarithm of the amount of sample copy number as the abscissa, which was calculated by the DNA concentration. The regression curve shows a linear relationship with a slope of −3.42 and a regression coefficient (R^2^) of 99.9% (Fig. 1).

### Crabapple seeds infected by with *V*. *mali*

Crabapple seeds were independently collected from four different orchards that were located in Zhangjiakou, Muyang, Lijiang and Baoding and had a history of AVC. Among the four sets of crabapple seeds, *V*. *mali* could be reliably quantified by qPCR, indicating that the infection of *V*. *mali* in crabapple seeds was common. Samples collected from Baoding and Muyang city had higher proportions of infected crabapple seeds than those collected from Zhangjiakou and Lijiang. The log concentration of *V*. *mali* gDNA in the infected seeds ranged from 2.15 to 2.49, without significant differences between the different regions (Table 2). In contrast, except for *Fusarium* spp., *Alternaria* spp. and *Penicillium* spp., no other fungi were isolated from Baling crabapple seeds collected from the above four different regions by traditional culture methods, which indicated that the qPCR assay is more sensitive for the detection of *V*. *mali* than traditional culture methods.

**Table 2:** Log concentration of *V*. *mali* in crabapple seeds and the proportion of infected seeds from different regions.

Regions	*N* ^a^	*LogC* ^b^	F	P	*p*_*s*_ (%)^c^
Zhangjiakou	117	2.15 ± 0.55	1.86	0.142	12.87
Muyang	67	2.49 ± 0.66			44.55
Lijiang	86	2.19 ± 0.66			22.19
Baoding	55	2.14 ± 0.61			49.01

^a^*N* means the number of crabapple seeds collected from different regions;

^b^*LogC* represents the log concentration of the *V*. *mali* genomic DNA copies in the infected crabapple seeds; the statistical analysis of *LogC* between different regions was performed with one-way analysis of variance (ANOVA), where P < 0.05 indicates significant difference;

^c^*p*_*s*_ indicates the proportion of crabapple seeds infected with *V*. *mali*.

### Evaluation of *V*. *mali* in crabapple seeds by high-throughput sequencing

Except for group H1, the results of high-throughput sequencing for *V*. *mali* were positive in groups H2, H3 and H4. In addition, the incidence of *V*. *mali* in group H2 and group H3 were 0.023% and 0.005% respectively. Due to artificial inoculation of *V*. *mali*, the relative abundance of *V*. *mali* in H4 was 2.977%, which is much greater than that in H2 and H3 (Table 3). The results of high-throughput sequencing for *Fusarium* spp., *Alternaria* spp., and *Penicillium* spp., which were isolated from crabapple seeds by traditional culture methods, were also positive for all four sample sets, and the relative abundance of these three genera was much higher than that of *V*. *mali*.

**Table 3:** Relative abundance of major fungi in crabapple seeds.

Fungus	Relative abundance (%)			
	H1	H2	H3	H4
*Fusarium* spp.	3.086	8.720	4.713	9.599
*Alternaria* spp.	2.443	0.566	0.578	0.253
*Penicillium* spp.	0.894	0.190	1.306	0.616
*Valsa* spp.	0.000	0.023	0.005	2.977

### Tissues infected with *V*. *mali* in crabapple seeds

To explore the infected tissues, the germinated crabapple seeds were dissected into exopleura, endopleura, cotyledon and plantule. *V*. *mali* was detected in the 4 different tissues by the qPCR assay. Interestingly, only 30% of exopleura and 20% of endopleura were infected with *V*. *mali*, and no *V*. *mali* was detected by qPCR in the cotyledons and plantules (Table 4). The density of *V*. *mali* gDNA in the exopleura was up to 1.12 × 10^3^ copies g^−1^, which was more than that in the endopleura (7.94 × 10^2^ copies g^−1^). This indicated that the infection tissues of *V*. *mali* in crabapple seeds are the exopleura and endopleura.

**Table 4:** The proportion of infected crabapple seed tissues and log concentrations of *V*. *mali* in infected tissues.

Seed tissue	N*	*p*_*s*_ (%)	LogC**	F	P
Exopleura	30	30	3.05 ± 0.11 a	10.01	0.007
Endopleura	30	20	2.90 ± 0.04 b		
Cotyledon	30	0	—		
Plantule	30	0	—		

*N indicates the number of crabapple seed tissues; *p*_*s*_ indicates the proportion of crabapple seeds infected with *V*. *mali*.

**LogC represents the log concentration of the *V*. *mali* genomic DNA copies in the infected crabapple seeds; “—” means the concentration of *V*. *mali* genomic DNA copies in sample is 0 copies g^−1^; the statistical analysis of *LogC* between different regions was performed with one-way analysis of variance (ANOVA); different letters indicate a significant difference between different crabapple seed tissues (P < 0.05).

### Development of *V*. *mali* in different growth periods of crabapple seedlings

**Figure 2:**
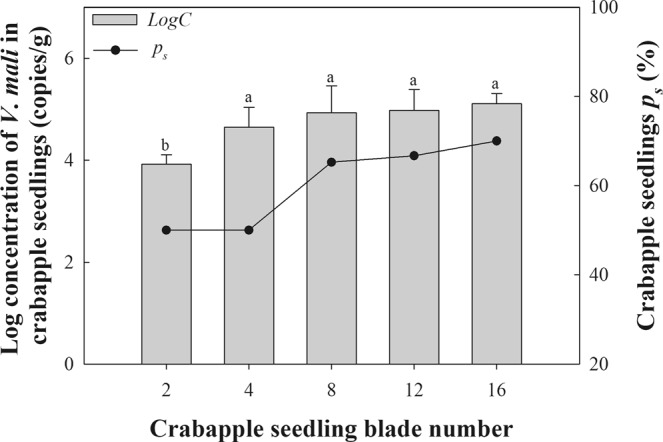
Development of *V*. *mali* in crabapple seedlings with the increase in blade number. Column diagram showing the log concentration of *V*. *mali* gDNA at different periods of crabapple seedlings (*LogC*). Line graph expressed the proportion of infected crabapple seedlings (*p*_*s*_). Error bars represent standard deviation from repeats. Different letters indicate a significant difference between different growth periods of the crabapple seedlings (P < 0.05).

The proportion of infected crabapple seedlings gradually increased with the growth of the seedlings. During the growth period of two, four, eight, twelve, and sixteen blades, 50.00%, 50.00%, 65.22%, 66.67%, and 70.00% of crabapple seedlings respectively, were infected with *V*. *mali*. In addition, the concentrations of *V*. *mali* gDNA in seedlings also significantly increased with the growth of crabapple seedlings. Only 8.32 × 10^3^ copies g^−1^, equivalent to 3.92 log copies, was detected in the infected seedlings during the period with two blades. When the seedlings had four blades, the log concentration of *V*. *mali* gDNA increased to 4.65, which was significantly higher than that in the second leaf period. After the four-leaf stage, with the increase in crabapple seedling leaves, the density of *V*. *mali* per unit weight of crabapple seedlings gradually increased, but there was no significant difference upon statistical analysis (Fig. 2).

### Infection of *V*. *mali* in one-year-old apple twigs and apple seeds

One-year-old apple twigs were stripped into phloem and xylem, which were used for *V*. *mali* detection individually. A total of 68.88% of phloem and 76.67% of xylem samples were positive for *V*. *mali* infection. Meanwhile, the log concentrations of *V*. *mali* gDNA in the infected apple trees remained at a high level of 4.35 in the phloem and 4.41 in the xylem, equivalent to 2.23 × 10^4^ copies g^−1^ and 2.57 × 10^4^ copies g^−1^, respectively, without a significant difference between the xylem and phloem (Table 5).

**Table 5:** *V*. *mali* infection in apple twig tissues and apple seeds.

Samples^a^	N^b^	*p*_*s*_ (%)^c^	*LogC* ^d^	t	P
**Apple twig tissue**					
Phloem	10	68.89	4.35 ± 0.42	−0.19	0.85
Xylem	10	76.67	4.41 ± 0.74		
**Apple seeds from**					
Healthy tree	27	0	—	−19.04	0.003
Diseased tree	27	11.11	3.18 ± 0.29		

^a^Phloem and xylem represent the phloem and xylem of apple twigs; Healthy tree seeds and diseased tree seeds represent the apple seeds collected from healthy trees or diseased trees;

^b^N indicates the total number of different tested apple twig tissues or tested apple seeds;

^c^*p*_*s*_ indicates the proportion of apple twig tissues or apple seeds infected with *V*. *mali*;

^d^*LogC* represents the log concentration of the *V*. *mali* genomic DNA copies in apple twig tissues or apple seeds; “—” indicates the concentration of *V*. *mali* genomic DNA copies in apple seeds from healthy trees is 0 copies g^−1^; the statistical analysis of *LogC* between different apple twig tissues or apple seeds was performed with independent sample T test, where P < 0.05 indicates significant difference.

To evaluate whether *V*. *mali* could be transferred from twigs to apple seeds, healthy or infected apple trees were surveyed and the seeds were gathered to detect *V*. *mali*. A total of 11.11% of seeds from infected apple trees with evident AVC symptoms were positive for *V*. *mali* infection, and the log concentration of *V*. *mali* was 3.18, equivalent to 1.51 × 10^3^ copies g^−1^ (Table 5). However, the test results of seeds from healthy apple trees without evident AVC symptoms were negative for *V*. *mali*. This result implied that *V*. *mali* in apple seeds may be transferred from infected apple twigs and become reservoirs of *V*. *mali* in old apple orchards.

## Discussion

AVC occurs not only in old orchards but also consistently in newly established orchards. Rootstocks, grafted branches, and apple seeds are suspected to serve as inoculum sources for AVC, especially in newly established orchards. Latent infection is an important feature of the AVC pathogen, and the concentration of *V*. *mali* gDNA in plants without clear symptoms of AVC is typically too low to be effectively detected by traditional culture methods due to background from complex microbial communities. To solve this problem, a qPCR assay was developed to detect the AVC pathogen for the first time. Using this method, crabapple seeds, crabapple seedlings, apple seeds and apple seedlings were shown to be capable of carrying the AVC pathogen, which may become reservoirs of *V*. *mali* in newly established apple orchards and old apple orchards.

Several different genes were explored as target sequences to design specific primers, and fortunately a suitable region was selected from the conserved translation elongation factor-1 α (EF1α) gene whose sequence exhibits a significant diversity among different *Valsa* species as previously described^17–19^. EF1α is a multimeric ribosomal protein that is ubiquitous and abundantly expressed in cells and involved in various important cell metabolism processes, including translational control, signal transduction, apoptosis, and cytoskeleton compositions^20–22^. The sequence of this gene, which is highly conserved and possesses the characteristics of housekeeping genes, has previously been employed in developing diagnostics for pathogenic fungi^23^. In this study, a partial sequence of the EF1α gene was selected to design a pair of species-specific primers for the detection of *V*. *mali*.

*V*. *mali* parasitizes apple tissues, such as apple bark and apple twigs, where the microbial community structure is complex. Hence, a highly specific primer is required to exclusively detect the target strain. In this study, sixteen *V*. *mali* strains from different regions, including the type strain 03–8, were used to evaluate the specificity of the primers. Meanwhile, a different pathovar of the same species (*V*. *mali* var. *pyri*), and twenty-one other strains of fungal species were consistently detected, including other pathogens of apple and saprophytic fungal species. The EF1α-based qPCR exclusively amplified all seventeen *V*. *mali* strains isolated from apple twigs in different regions with no amplification for the twenty-one other reference strains. This indicated that the newly designed primers are species specific for *V*. *mali*. A 10-fold dilution series of standard samples covering the range 0.2–2 × 10^7^ *V*. *mali* gDNA copies per reaction volume was used to estimate the limit of quantification^24^. The lowest amount of gDNA that produced replicates with a CV ≤ 35 was 2 gDNA copies, which indicated that the limit of quantification was 2 genome copies. This result is consistent with previous studies of *Magnaporthe oryzae*^12^, *Pseudomonas cichorii*^25^, *Pseudomonas syringae* pv. *lachrymans*^14^ and *Sclerotinia sclerotiorum*^26^. However, for pathogens in plant tissues, detection by qPCR would be not as easy as detection from pure culture because PCR inhibitors may restrict the process of qPCR^27^. Thus, effectively removing PCR inhibitors during DNA extraction is the key to detecting the pathogen in plant tissues. In this study, a modified CTAB method was employed to prevent DNA degradation and facilitate DNA precipitation during DNA extraction, which improved DNA purity^11^.

Thus far, whether rootstocks carry the AVC pathogen and AVC spreads to apple branches remain unclear. In this study, qPCR assays were used to evaluate the presence of *V*. *mali* in crabapple seeds and seedlings. Our results indicated that the infection of crabapple seeds by *V*. *mali* was common, although the proportions of infected seeds among the total seeds were different in different regions. Compared with the traditional method, the qPCR assay was more sensitive. Except for *Fusarium* spp., *Alternaria* spp. and *Penicillium* spp., the AVC pathogen could not be isolated from crabapple seeds using traditional culture methods, but it was detected in crabapple seeds using the qPCR assay. This may be due to the low relative abundance of *V*. *mali* in latent-infected crabapple seeds. This speculation was confirmed by the results of high-throughput sequencing, which revealed that *Fusarium* spp., *Alternaria* spp. and *Penicillium* spp. are the three most abundant fungi with high relative abundances in crabapple seeds, and the abundance of *Valsa* spp. was relatively low in crabapple seeds.

In addition, the exopleura and endopleura were only two infected tissues of *V*. *mali* in crabapple seeds, and the density of *V*. *mali* in the exopleura was greater than that in the endopleura. Obviously, the infection of *V*. *mali* starts from the outside of the crabapple seed. *V*. *mali* penetrates beyond the exopleura by an unknown mechanism but may be blocked by the endopleura. This phenomenon was also observed in some symbiotic fungi^28^. For instance, a symbiotic fungus in *Calluna* cannot penetrate beyond the exopleura^29^, but another fungus in *Lolium* can enter the plantule and penetrate beyond the growing point^30^. However, the mechanism of this phenomenon remains unknown and requires further research.

Although the density of *V*. *mali* in crabapple seeds was relatively low, the content of the pathogen increased rapidly with the development of the crabapple seedlings until eight or more blades emerged. As expected, the content of the AVC pathogen was maintained at a relatively high level in the crabapple seedlings with eight and sixteen blades and was 100 times more than that in seedlings in the two blade stage. This indicated that although the content of *V*. *mali* in the latent infection stage was relatively low in crabapple seeds, it increased rapidly in a suitable environment. Thus, the infected crabapple seeds or seedlings may play an important role as a primary source of *V*. *mali* infection in newly established orchards.

Apple twigs were gathered from a scion nursery and used to detect *V*. *mali* whose mycelia may invade the phloem and even deeper apple tissues such as the xylem^31^. As expected, most of the tested twigs from the scion nursery were positive for *V*. *mali*, in both phloem and xylem, and the density of *V*. *mali* remained relatively high in these tissues. This indicated that the apple twigs latently infected with *V*. *mali* and used as grafted branches have a risk of being potential inoculum sources in newly established orchards.

To determine whether the AVC pathogen can be transferred from twigs to apple seeds, apple seeds gathered from healthy trees or infected trees were used to detect *V*. *mali* by qPCR. Approximately ten percent of the apple seeds from infected trees were positive for *V*. *mali* detection, while none of the healthy trees harboured the pathogen. This indicated that the AVC pathogen in apple seeds may be transferred from infected twigs, and the infected apple fruits left in apple orchards may become a potential reservoir of *V*. *mali* in the following year.

## Materials and Methods

### Fungal isolation

Field surveys were conducted in apple orchards from different apple producing regions in China, and apple twigs with typical canker symptoms were collected to isolate the AVC pathogen. The apple twigs with lesions were cut off at the edge of the infected area, surface sterilized with 75% ethanol for 2 to 3 min, and transferred to potato dextrose agar (PDA) for incubation at 25 °C for three days. The fresh mycelia were transferred to new PDA to purify the pathogen. Single-spore isolation was conducted according to a previous method^32^. Three isolates were identified as *V*. *mali* according to pathogenicity, morphology and ITS sequence analysis^4^. The isolates and reference strains, which are listed in Table 1, were stored in 15% glycerol at −80 °C refrigerator in College of Plant Protection, Hebei Agricultural University (HAU).

### DNA extraction

Colony plugs (5 mm in diameter) of the isolates were transferred from potato dextrose agar into 25 ml aliquots of potato dextrose broth. The cultures were incubated at 25 °C for seven days with shaking at 100 rpm. Approximately 0.1 grams of fungal mycelia was lyophilized and ground into powder in liquid nitrogen. Genomic DNA of pure culture strains was extracted by the CTAB method^33^. The plant tissues were frozen in liquid nitrogen, and ground into powder with a pre-cooled mortar and pestle. Fungal DNA extraction from plant tissues was performed using an optimized CTAB method^11^. The DNA was dissolved in 100 µL nuclease-free water and preserved at −20 °C. The extracted DNA concentration was determined using a NanoDrop 2000/2000c (Thermo Fisher Scientific, USA). The DNA concentration of pure culture fungi ranged from 800 ng per µl to 1500 ng per µl, and the DNA concentration of plant tissues ranged from 500 ng per µl to 1000 ng per µl. DNA was diluted to the required concentration using nuclease-free water.

### Primer design and specificity

**Figure 3:**
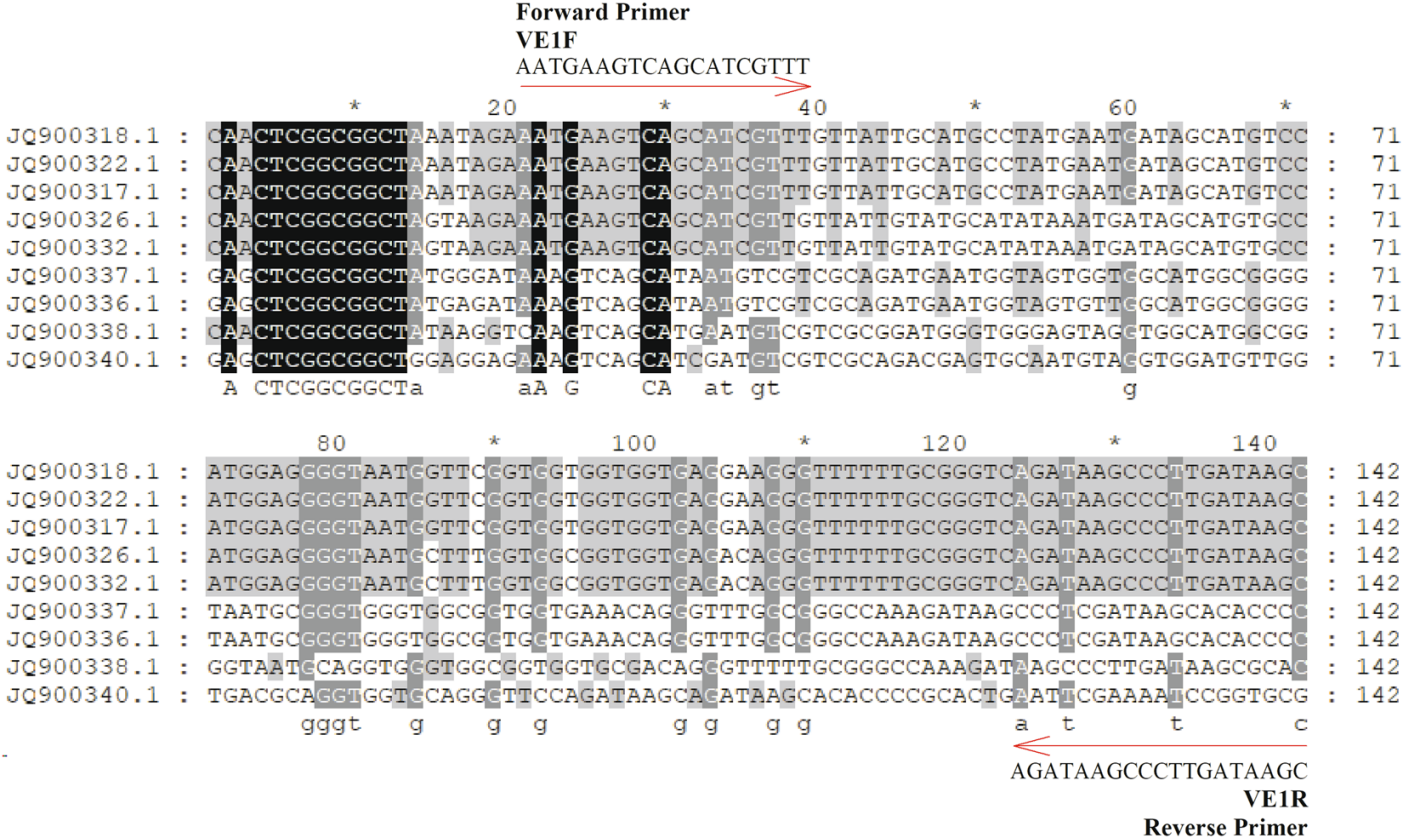
Multiple sequence alignment of the 142 bp partial eukaryotic elongation factor 1 alpha (EF-1ɑ) gene sequences. Polymorphic sites were used to design specific primers VF1F/VE1R for *V*. *mali*. The accession numbers JQ900318.1, JQ900322.1 and JQ900317.1 are the partial EF-1ɑ genes of *Valsa mali* var. *mali*; JQ900326.1 and JQ900332.1 are the partial EF-1ɑ genes of *Valsa mali* var. *pyri*; JQ900337.1 and JQ900336.1 are the partial EF-1ɑ genes of *Valsa malicola*; JQ900338.1 is the partial EF-1ɑ gene of *Cytospora chrysosperma*; and JQ900340.1 is the partial EF-1ɑ gene of *Leucostoma persoonii*. The primer sequences are indicated (VE1F as a forward complement and VE1R as a reverse complement). Conserved sequences between the *Valsa mali* var. *mali* strains are shaded in grey, and mismatches with other species or genus strain sequences are indicated with dark shading.

Based on sequence alignments of the translation elongation factor-1 α (EF1α) gene from three *V*. *mali* var. *mali* strains and six other species or genus strains available in the NCBI database (Fig. 3), the primer pair VE1-F (5′-AAT GAA GTC AGC ATC GTT T-3′) and VE1-R (5′-GCT TAT CAA GGG CTT ATC T-3′) was designed for specific amplification of *V*. *mali* DNA.

To confirm the specificity of the primer pairs, sixteen *Valsa mali* strains, including the type strain 03–8 that was donated from Prof. Huang in Northwest A&F University^34^ and twenty-two reference strains were prepared for DNA extraction. The strains used in this study are stored at HAU. After DNA extraction, the DNA of the strains was diluted to 10 ng per microlitre and used for qPCR detection (Table 1). The amplification products of different *V*. *mali* strains were re-sequenced, and the identity of the sequences was checked against the NCBI database using the basic local alignment search tool (http://blast.ncbi.nlm.nih.gov/Blast.cgi).

### Real-time PCR conditions

Prior to amplification, the optimal concentration of specific primers (100 to 700 nM final concentration) and gradient PCR (50 °C to 62 °C) were analysed to obtain an optimum final primer concentration (200 nM final concentration) and annealing temperature (60 °C) for the qPCR assay. qPCR was conducted in a 20 µl reaction volume containing 1 µl template, 10 µl of 2 × FastStart Essential DNA SYBR Green Master (Roche Diagnostics GmbH, Germany), and 1 µl of each primer (VE1-F and VE1-R). Amplification was performed in the LightCycler^®^ 96 Real-Time PCR System (Roche Molecular Systems, Inc., Germany) with an initial denaturation step at 95 °C for 5 min, followed by 40 cycles at 95 °C for 12 s, 58 °C for 12 s, and 72 °C for 14 s. A melting curve analysis was performed from 60 °C to 95 °C, with 0.5 °C/30 s increments. Quantification cycles (Ct values) were obtained automatically by LightCycler^®^ 96 Software (Version 1.1.0.1320), and qPCR of each template was repeated three times.

### Limit of quantification and quantitative determination

The 10-fold serial dilutions of genomic DNA were used to test the limit of quantification covering the range 2 to 10^7^ gDNA copies per reaction volume. Each standard genomic DNA was analysed in ten replicates. The standard deviation (SD) of each replicate sample at the different concentrations was calculated with Ct values that reflected the average difference of the measured values to the mean on the same scale. The coefficient of variation (CV) was calculated with the SD and the mean values of gDNA copy number (CV = 100 × SD/mean). The limit of quantification was specified as the lowest concentration at which replicates had a CV ≤ 35% at the calculated concentration^24^.

In addition, a standard curve for the quantification of *V*. *mali* was established using serial 10-fold dilutions of the genomic DNA, ranging from 264 ng to 2.64 × 10^−7^ ng. The standard curve was plotted as the logarithm of the sample copy number versus the quantification cycles (Ct values). The sample copy number was calculated with the following equation^14,25^:

$$N\,=\,\frac{C\times AN}{n\times mw}$$

where *N* is the sample copy number, *C* is the concentration of DNA template, *AN* is Avogadro’s number (6.023 × 10^23^ molecules mol^−1^), *n* is the genome size of *V*. *mali* (44.7 Mb), and *mw* is the average molecular weight per bp (660 g mol^−1^). The detection of 10-fold serial dilutions of *V*. *mali* DNA was repeated three times and each DNA template was performed three times to establish the standard curve.

### Detection of *V*. *mali* in crabapple seeds from different regions

Four sets of Baling crabapple seeds were collected from Zhangjiakou city, Muyang city, Lijiang city, and Baoding city in China. The seeds were first surface sterilized by soaking them in 0.1% sodium hypochlorite for ten min and 70% ethyl alcohol for 2 min, and then the seeds were washed with sterile water three times. After drying, each seed was separately ground into powder to extract the total genomic DNA using the CTAB method^11^. Seed genomic DNA was used to detect *V*. *mali* by qPCR as described previously. The samples were considered to be infected with *V*. *mali* if the Ct values were less than 34.4. The concentration of *V*. *mali* gDNA in each infected seed was calculated by the Ct value according to the formula described previously. The log transformation of DNA concentration was performed, and the log concentration of *V*. *mali* in the crabapple seeds was statistically analysed using one-way analysis of variance. The proportion of infected crabapple seeds (*p*_*s*_) was calculated by the following equation.

$${p}_{s}=\frac{{N}_{i}}{{N}_{t}}\times 100 \% $$

where *N*_*i*_ represents the infected seed number, and *N*_*t*_ is the total number of crabapple seeds.

### Detection of *V*. *mali* in crabapple seed tissues

**Figure 4:**
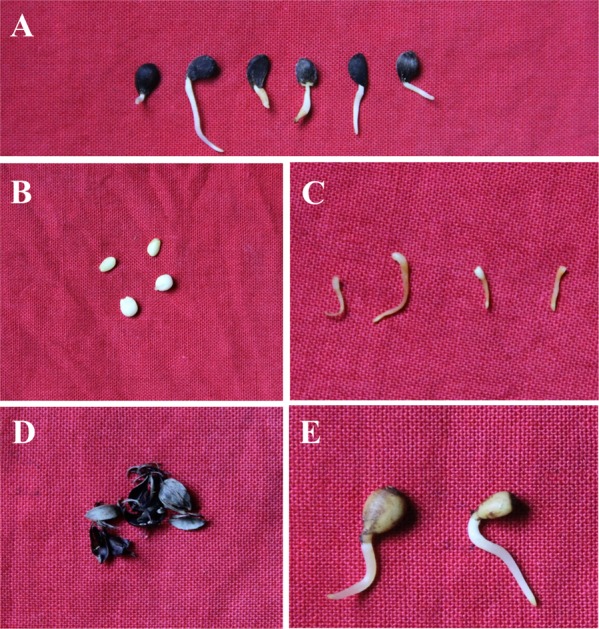
Different tissues of crabapple seeds. The germinated seeds (**A**) are composed of cotyledon (**B**), plantule (**C**), exopleura (**D**) and endopleura (**E**).

Thirty seeds collected from Zhangjiakou city were used to analyse the infected tissues in crabapple seeds. All of the seeds were subjected to germination with the following process. All of the crabapple seeds were incubated at 37 °C for 12 h and then mixed with sterilized sand at a mass ratio of 1:5. The mixtures were incubated in wet sand at 4 °C for three months. Then, the crabapple seeds were incubated at 37 °C for 18 h in a water bath and transferred to 4 °C while covered with four layers of wet gauze for a week to germinate. The germinated crabapple seeds were surface sterilized as described above. Each seed was striped separately into four different tissues with a sterilized dissecting needle, including exopleura, endopleura, cotyledon and plantule (Fig. 4), and each tissue was individually ground into powder to extract the total genomic DNA using the CTAB method. The total genomic DNA of each tissue was used to detect *V*. *mali* by qPCR. The concentration of *V*. *mali* gDNA in infected tissue was calculated with the Ct value according to the formula described previously. The log transformation of the DNA concentration was performed, and the log concentration of *V*. *mali* was statistically analysed using one-way analysis of variance. The proportion of infected crabapple seed tissues (*p*_*s*_) was also calculated as mentioned above.

### Evaluation of seeds carrying *V*. *mali* by high-throughput sequencing

To confirm that *V*. *mali* was carried by the crabapple seeds, high-throughput sequencing was employed to analyse the microbial diversity of the crabapple seeds. Forty surface-sterilized crabapple seeds were equally divided into four groups, labelled H1, H2, H3 and H4, and those of H4 were soaked in 10 µL *V*. *mali* spore suspension at a concentration of 10^7^ CFU/ml before DNA extraction. Total genomic DNA was extracted by the CTAB method as described previously and the DNA samples were sent to Shanghai Personal Biotechnology Co., Ltd. (Shanghai, China) for high-throughput sequencing. The sequences were aligned, and the operational taxonomic units (OTUs) were divided by QIIME software using the UCLUST sequence alignment tool^35^. The taxonomy information of each OTU was obtained by aligning the representational sequence of OTUs with the corresponding database.

### Detection of *V*. *mali* in crabapple seedlings

Two hundred germinated crabapple seeds collected from Zhangjiakou were planted in a greenhouse at HAU at 30 °C/25 °C for day and night temperatures. Seedlings with two, four, eight, twelve, and sixteen blades were collected and ground into powder in liquid nitrogen after surface sterilization. Each seedling was prepared for DNA extraction and *V*. *mali* detection by qPCR as previously described. The concentration of *V*. *mali* gDNA in infected seedlings was calculated by the Ct value according to the formula described previously. The log transformation of the DNA concentration was performed, and the log concentration of *V*. *mali* in the crabapple seedlings was statistically analysed using one-way analysis of variance. The proportion of infected crabapple seedlings (*p*_*s*_) in each group was calculated as mentioned previously.

### Detection of *V*. *mali* in apple twigs

A survey was conducted at a scion nursery in Li county, Baoding city, Hebei Province, China, in June 2016. Thirty one year-old twigs were gathered from ten randomly selected apple trees. After being surface sterilized, the apple twigs were stripped into two different tissues, xylem and phloem, using dissecting needles. Each apple twig tissue of 0.15 grams was subjected to DNA extraction and qPCR assays separately. The concentration of *V*. *mali* gDNA in infected apple twig tissues was calculated by the Ct value according to the formula as described previously. The log transformation of the DNA concentration was performed, and the log concentration of *V*. *mali* in the phloem and xylem was statistically analysed using an independent sample T test. The proportion of infected apple twig tissues (*p*_*s*_) was calculated as abovementioned.

### Detection of *V*. *mali* from apple seeds by qPCR

A survey was conducted in three apple orchards in Baoding city, Hebei Province, China in November 2016. Two sets of apple seeds were collected either from healthy apple trees or from diseased trees. Each seed was surface sterilized as described previously, and then ground into a powder to extract genomic DNA by the CTAB method^11^. Genomic DNA of each apple seed was tested for *V*. *mali* by the qPCR assay as previously described. The concentration of *V*. *mali* gDNA in each infected apple seed was calculated by the Ct value according to the formula described previously. The log transformation of the DNA concentration was performed, and the log concentration of *V*. *mali* was statistically analysed using an independent sample T test. The proportions of infected apple seeds (*p*_*s*_) were calculated as described above.

### Statistical analysis

The data of the *V*. *mali* gDNA concentrations were log transformed into normalized data. The log concentrations were tested for normality and homoscedasticity before statistical analysis by one-way analysis of variance (ANOVA) or independent sample T test according to the factorial design by using SPSS V.24 software (IBM SPSS Statistics Version 20, Somers, NY, USA). The least significant difference tests were used to compare different treatment data at the 5% significance level by LSD method or T test. The proportions of infected seeds or tissues were calculated by Microsoft Excel 2010, and the statistical analysis was performed with the chi-square test using SPSS V.24 software (IBM SPSS Statistics Version 20, Somers, NY, USA).

## Data Availability

All data generated or analysed during this study are included in the main text of this article, and the raw data are available from the corresponding author.
